# New Insights in Cytokines in Childhood Obesity: Changes in TWEAK and CD163 After a 2-Year Intervention Program in Prepubertal Children With Obesity

**DOI:** 10.3389/fendo.2022.909201

**Published:** 2022-07-08

**Authors:** Rocío Escartín, Maria Font, José Miguel González-Clemente, Joan Vendrell, Assumpta Caixàs, Raquel Corripio

**Affiliations:** ^1^Pediatric Endocrine Department, Hospital Universitari Parc Taulí, Institut d’Investigació i Innovació Parc Taulí, Universitat Autònoma de Barcelona, Sabadell, Spain; ^2^Endocrinology and Nutrition Department, Hospital Universitari Parc Taulí, Institut d’Investigació i Innovació Parc Taulí, Universitat Autònoma de Barcelona, Sabadell, Spain; ^3^Endocrinology and Diabetes Unit, Research Department, Hospital Universitari Joan XXIII de Tarragona, Institut Pere Virgili, Tarragona, Spain; ^4^CIBER de Diabetes y Enfermedades Metabólicas Asociadas (CIBERDEM), Instituto de Salud Carlos III, Madrid, Spain

**Keywords:** TWEAK, CD163, childhood, obesity, prepubertal

## Abstract

**Objective:**

Obesity is characterized by a low-grade inflammatory state in adipose tissue. Tumor Necrosis Factor Weak Inducer of Apoptosis (TWEAK) and Cluster of Differentiation 163 (CD163) are cytokines potentially involved in the pathogenesis of obesity. Little is known about them in children. The aim of this study was to observe serum levels of TWEAK and CD163 in prepubertal children with obesity compared to lean, and to evaluate its changes after a 2-year intervention program in children with obesity.

**Methods:**

Case-control study with a prospective follow-up of cases for 2 years in a referral pediatric endocrine outpatient centre. Seventy-three prepubertal children with obesity, and forty-seven age- and gender-matched lean controls were studied. Sixty-two cases finished the program. Anthropometric parameters, Homeostatic Model Assessment for Insulin Resistance (HOMA-IR), lipid profile, and concentrations of TWEAK and CD163 were determined. Children with obesity were re-evaluated after a 2-year intervention program consisting of diet and exercise. Weight loss was considered if z-score Body Mass Index (BMI) decreased at least 0.5 Standard Deviations (SD).

**Results:**

We observed higher CD163 levels in children with obesity compared to controls. No significant differences were observed in TWEAK and CD163/TWEAK ratio at baseline. After the 2-year intervention program, TWEAK levels were higher and CD163/TWEAK ratio was lower in children with weight loss than those without weight loss. CD163 decreased in both groups.

**Conclusion:**

TWEAK and CD163 seem to have a role in the pathogenesis of obesity in prepubertal children.

## Introduction

The prevalence of childhood obesity has increased dramatically in recent decades, becoming a global public health problem (1). Pathological conditions associated with obesity, such as diabetes, hyperlipidemia, or hypertension (2), have been observed mainly in adults. As the prevalence of childhood obesity increases, these conditions are becoming more common among infants and teenagers (3–8).

Obesity is associated with a chronic inflammatory state in adipose tissue (9–11). In this context, we can observe altered levels of molecules involved in the regulation of inflammation, called cytokines (12–14).

Research on cytokines has increased during the last years in order to identify risk and protective factors of cardiovascular disease and to generate potential target treatments (15, 16).

Tumor Necrosis Factor Weak Inducer of Apoptosis (TWEAK), also known as Tumor Necrosis Factor (TNF) ligand superfamily member 12, is a cytokine involved in multiple biological functions (12, 17, 18). It seems to play a protective role in the regulation of obesity and insulin resistance (15, 19, 20). It was observed that serum levels of soluble TWEAK are lower in adults with severe obesity, and they increase in these patients after a significant weight loss (21).

Cluster of Differentiation 163 (CD163) is a macrophage-specific protein that increases in the context of inflammation (22). *In vitro*, it has been identified as a scavenger receptor of TWEAK, promoting its degradation (23). Serum levels of CD163 are higher in obesity and insulin resistance, being a strong predictor of type 2 diabetes in adults (24). In cardiovascular diseases, a high CD163/TWEAK ratio was found (25).

Little is known about TWEAK and CD163 in children. The main objective of the study was to evaluate if a significant weight loss in children with obesity may induce changes in these parameters after a 2-year lifestyle intervention program. In addition, the investigation aimed to evaluate serum levels of TWEAK, CD163, and CD163/TWEAK ratio in prepubertal children with obesity compared to lean children.

## Patients and Methods

Seventy-three prepubertal children with obesity were included, from an outpatient long-term intervention program over a period of 2 years. Inclusion criteria were the presence of obesity as defined by body mass index (BMI) > 2 standard deviation (SD) scores for age and sex upon Spanish normative charts (26), aged between 6 and 10 years and prepubertal according to Tanner staging (27, 28). Controls were forty-seven prepubertal healthy children attending for preoperative blood tests before minor surgery. They were matched by age and gender. Exclusion criteria were the presence of endocrinopathies, obesity-associated syndrome, and any infectious or inflammatory disease in the past 10 days, or taking medication that affected weight, lipid metabolism, or arterial blood pressure (BP). Written informed consent was obtained from all patients’ parents, and all investigations followed the Helsinki Declaration. The study was approved by the Ethics Committee of our Institution (reference code 2004104).

### Baseline Clinical Evaluation

Detailed medical, personal, and family history of obesity and cardiometabolic risk was obtained from all subjects, including birth weight and length for gestational age. A complete physical examination was performed. Height was measured by a Harpenden stadiometer to the nearest 0.1 cm and body weight by balance scale to the nearest 0.1 kg. Waist circumference was measured with a tape at the middle point between the last rib and the superior iliac crest, adjusted to the nearest 0.1 cm, and compared with an age and sex-reference population (29). All measurements were performed for duplication by the same investigator, with the patient in light clothes and without shoes. The mean of the two determinations was used for calculations. Adiposity was evaluated by BMI (calculated as weight in kilograms divided by the square of height in meters). Pubertal development was assessed by direct physical examination according to Tanner staging. Blood pressure was measured by triplicate with the Critikon Dinamap 8100 automatic system (Johnson-Johnson Company, Tampa, FL, USA), with an appropriate sized cuff and after at least ten minutes resting in supine position. The lowest BP value was recorded and evaluated using the percentiles of the International Task Force for Blood Pressure (30).

Obesity degree was fixed with z-BMI using the LMS method (31). We also applied the obesity criteria by Cole (International Obesity Task Force) (32) to our BMI data.

### Baseline Metabolic Evaluation

Blood samples were obtained after a 12-hours overnight fast. Concentrations of soluble TWEAK, soluble CD163, alanine aminotransferase (ALT), uric acid, glucose, insulin, cholesterol and triglycerides were determined. Likewise, a standard 2-hours oral glucose tolerance test was performed in subjects with obesity.

Samples were stored at -80°C until their analysis. Plasma glucose was measured by the glucose hexokinase method, insulin by an electrochemiluminescent method, cholesterol and its fractions by cholesterol esterase/oxidase, and triglycerides by lipase/glycerol kinase (Roche Diagnostics, Mannheim, Germany). Intra- and inter-assay coefficient of variation (CV) values were 1.9–2.1% for glucose and 2.6–2.8% for insulin, respectively.

Plasma soluble TWEAK was measured by an enzyme-linked immunoassay (ELISA) technique with a sensitivity of 0.02 pg/mL (intra-assay CV <10%, inter-assay CV <9%). Plasma soluble CD163 was measured by ELISA with a sensitivity of 0.1 ng/mL (intra-assay CV <6%, inter-assay CV <7%). Impaired glucose metabolism was defined according to the American Diabetes Association criteria (33). Insulin resistance was evaluated using the Homeostatic Model Assessment for Insulin Resistance $\left[HOMA\;IR=\frac{fasting\;insulin\;\left(µU/mL\right)*fasting\;glucose\;\left(µmol/L\right)}{22,5}\right]$ (34).

### Intervention and Follow-Up

After the baseline evaluation, 73 prepubertal children with obesity started a lifestyle intervention program that included a balanced norm caloric diet adjusted by age and a personally adapted exercise program. The diet contained 30% of energy intake from fat, 15% from protein, and 55% from carbohydrate (5% as sugar). A plan of 30–45 minutes of moderate exercise three times a week was negotiated. Television and video games were limited to a maximum of 2 hours a day. Follow-up visits were scheduled every 4 months.

After 2 years, a clinical and metabolic evaluation was performed with the same parameters as in the baseline evaluation. We considered a significant weight loss if the z-score of BMI had decreased at least 0.5 SD (35).

### Statistical Analysis

Data were expressed as mean ± SD for quantitative variables and as percentages for categorical variables. Logarithmic transformation before the analysis was used when variables did not follow a normal distribution. Student’s t-test or Mann-Whitney U test were used for comparing differences between groups. Paired t-test or Wilcoxon test was used to compare variables before and after the intervention program. Bivariate correlations were evaluated with Pearson’s and Spearman’s coefficients as appropriate. Furthermore, some multivariate linear regression models were used. TWEAK, CD163 or CD163/TWEAK ratio as dependent variables and age, sex, weight status (BMI), HOMA-IR and lipid profile, as independent variables. A p value < 0.05 was considered significant. Analyses were performed with SAS v9.4, SAS Institute Inc., Cary, NC, USA.

## Results

73 children with obesity and 47 controls were included in the study. Characteristics of subjects are shown in **Table 1**. Comparing cases and controls, CD163 levels were higher in children with obesity. No statistically significant differences were found in TWEAK and CD163/TWEAK ratio.

**Table 1:** Baseline Subjects Characteristics.

	With obesity (n = 73)	Control (n = 47)	*p*
**Sex**	37 girls/36 boys	16 girls/31 boys	0.073
**Age (years)**	8.03 ± 1.08	7.74 ± 1.35	0.22
**BMI (Kg/m^2^)**	26.5 ± 3.07	16.2 ± 1.41	<0.001
**z-BMI (SD)**	4.76 ± 1.67	-0.27 ± 0.74	<0.001
**Birth Weight (gr)**	3337 ± 599.5	3194 ± 539.9	0.28
**Birth Length (cm)**	50.0 ± 2.61	50.1 ± 1.60	0.3
**Familiar diabetes 2**	35 (47%)	11 (23%)	0.01
**Father BMI (Kg/m^2^)**	28.6 ± 3.93	25.2 ± 2.31	0.003
**Mother BMI (Kg/m^2^)**	29.1 ± 7.83	23.1 ± 3.22	0.001
**Acanthosis**	23 (31%)	0 (0%)	<0.001
**Waist (cm)**	81.3 ± 8.5	57.5 ± 6.05	0.035
**SBP (mmHg)**	109.2 ± 14.5	104.1 ± 9.60	0.026
**DBP (mmHg)**	63.4 ± 8.32	64.1 ± 9.25	0.672
**Glucose (mg/dL)**	83.7 ± 7.78	83.3 ± 5.04	0.804
**Insulin (μU/mL)** ^a^	10 (5-15)	4 (2-5)	<0.001
**HOMA-IR** ^a^	1.97 (1.02-3.14)	0.80 (0.40-1.08)	<0.001
**Cholesterol (mg/dL)**	155.5 ± 27.9	157.8 ± 31.4	0.621
**Triglycerides(mg/dL)** ^a^	69 (52.5-87.5)	48 (40-54)	<0.001
**LDLc (mg/dL)**	93.5 ± 24.1	82.6 ± 24.7	0.032
**HDLc (mg/dL)**	51.3 ± 12.8	65.5 ± 14.7	<0.001
**Uric acid (mg/dL)**	4.12 ± 0.85	3.41 ± 0.626	<0.001
**ALT (U/L)**	20.1 ± 6.68	19.2 ± 6.97	0.479
**TWEAK (pg/mL)**	1479.1 ± 1347.7	1184.9 ± 771.8	0.244
**CD163 (ng/mL)**	210.2 ± 48.9	187.2 ± 31.6	0.012
**CD163/TWEAK ratio**	0.14 ± 0,07	0.15 ± 0,10	0.255

Data are presented as mean ± SD or percentages.

ALT, alaninetransferase; BMI, body mass index;CD163, Cluster of differentiation 163; DBP, diastolic blood pressure; HDLc, high density lipoprotein cholesterol; HOMA-IR, homeostatic model insulin resistance index; LDLc, low density lipoprotein cholesterol; SBP, systolic blood pressure; TWEAK, Tumor Necrosis Factor weak inducer of apoptosis.

a Median (interquartile range).

**Figure 1:**
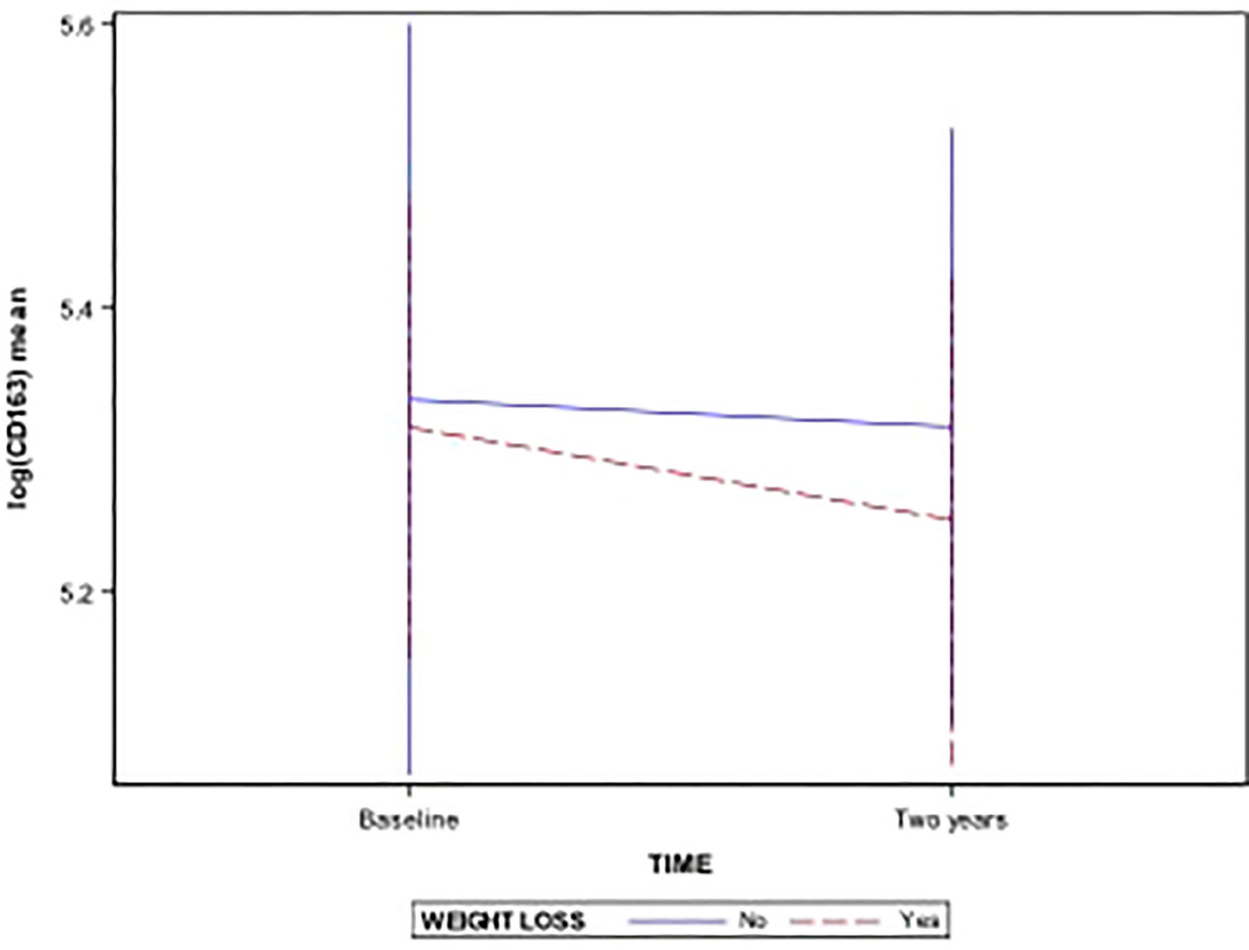
CD163 decreased after 2 years in both groups, and the decrease was more pronounced in patients with weight loss.

**Figure 2:**
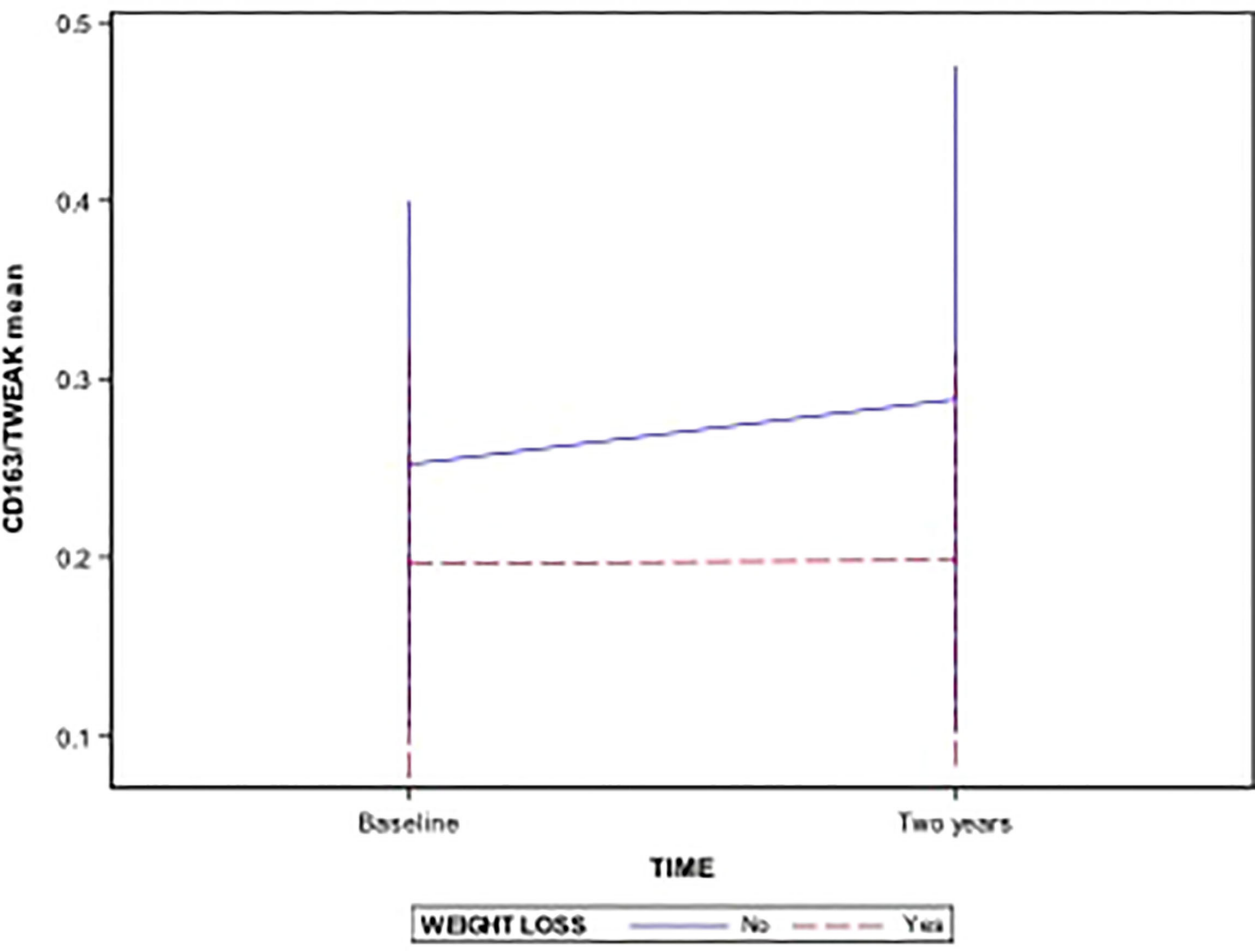
CD163/TWEAK ratio was higher in children without weight loss, and it increased after 2 years in this group.

After a 2-year follow-up of the cases, 62 completed the study and 11 dropped out (15%). 31 patients achieved a significant weight loss. Comparing patients with and without weight loss after the intervention program, TWEAK decreased in both groups after the intervention program. However, we observed that this parameter was higher in patients who achieved weight loss, before and after the intervention program. Serum levels of CD163 decreased after 2 years in both groups, and the decrease was more pronounced in patients with weight loss (**Figure 1**). CD163/TWEAK ratio was higher in children without weight loss, and it increased after 2 years in this group (**Figure 2**). Data are shown in **Table 2**.

**Table 2:** Baseline and after 2-years follow up characteristics according to weight loss in children with obesity.

	Without weight loss (n = 31)		With weight loss (n = 31)		
Sex	16 girls/15 boys		16 girls/15 boys		
	Baseline	Two years	Baseline	Two years	*p* ^a^
**Age (years)**	7.6 ± 1.0	9.9 ± 1.1 ^d^	8.4 ± 1.0^c^	10.4 ± 1.1 ^d^	
**BMI (Kg/m^2^)**	26.0 ± 2.7	28.8 ± 3.4 ^d^	26.5 ± 2.8	25.9 ± 3.3 ^d^	<0.001
**z-BMI(SD)**	4.68 ± 1.63	5.0 ± 1.7 ^d^	4.6 ± 1.3	3.3 ± 1.3 ^d^	<0.001
**Waist (cm)**	78.4 ± 7.1	87.5 ± 10.6^d^	81.5 ± 6.4 ^c^	83.6 ± 8.2	0.002
**SBP (mmHg)**	111.8 ± 16.1	111 ± 8.8	107 ± 12.4	108 ± 11.4	0.773
**DBP (mmHg)**	64.1 ± 8.6	69.5 ± 9.6	61.9 ± 8.8	61.7 ± 9.1	0.252
**Glucose (mg/dL)**	83.9 ± 6.7	88.5 ± 7.2^d^	84.2 ± 7.57	88.4 ± 8.87 ^d^	0.829
**Glucose 120min**	110 ± 21.5	112 ± 16.7	112 ± 16.7	110 ± 14.9	0.394
**Insulin (μU/mL)** ^b^	9 (5-13.2)	15 (10-20.2) ^d^	10 (5-13)	11 (8-14)	0.374
**HOMA** ^b^	1.9 (1.0-2.7)	3.3 (2.0-4.6) ^d^	1.9 (0.9-2.7)	2.5 (1.8-3.1) ^d^	0.371
**Cholesterol (mg/dL)**	155 ± 32.1	160 ± 30.1	157 ± 25.5	161 ± 28.1	0.907
**Triglycerides (mg/dL)** ^b^	62 (53-76)	77 (61-89) ^d^	72 (44-15)	75 (58-108)	0.437
**LDLc (mg/dL)**	92.1 ± 29.1	92.2 ± 28.6	95.8 ± 19.6	94.8 ± 20.3	0.685
**HDLc (mg/dL)**	55.1 ± 12.4	53.2 ± 12.9	49.8 ± 13.5	50.7 ± 15.2	0.506
**Uric acid (mg/dL)**	4.26 ± 0.80	4.68 ± 0.87 ^d^	3.83 ± 0.82 ^c^	4.12 ± 0.75	0.932
**TWEAK (pg/mL)**	1081.5 ± 699.6	1017.7 ± 720.7	1792.3 ± 1637.0	1535.9 ± 1370.4	0.032
**CD163 (ng/mL)**	215.0 ± 61.36	208.3 ± 49.2	206.2 ± 36.1	193.4 ± 34.1	0.334
**CD163/TWEAK ratio**	0.252 ± 0.148	0.289 ± 0.187	0.197 ± 0.120	0.198 ± 0.115	0.032

Data are presented as mean ± SD.

a Mean of % of change between both groups. % of change= (end value - initial value)/initial value x 100.

b Median (interquartile range).

c Baseline comparison between those with and without weight loss (independent t-test) (p<0.05).

d Two year paired t-test (p<0.05).

BMI, body mass index; CD163, Cluster of differentiation 163; DBP, diastolic blood pressure; HDLc, high density lipoprotein cholesterol; HOMA-IR, homeostatic model insulin resistance index; LDLc, low density lipoprotein cholesterol; SBP, systolic blood pressure; TWEAK, Tumor Necrosis Factor Weak Inducer of Apoptosis.

To further understand the relationship between TWEAK and CD163 and the rest of the variables, we carried out some bivariate correlation analysis with each of them. Age, sex, HOMA, and lipid profile were included as independent variables. No significant correlation among all these variables was found.

Forty-nine percent of the patients began puberty (assessed by Tanner stage) during the follow-up phase. After adjusting the regression model by Tanner, results were not modified.

## Discussion

TWEAK and CD163 have been linked to obesity and associated cardiovascular diseases in adults (15). However, there are still few data about these cytokines in children.

Our investigation did not find statistically significant differences in TWEAK or CD163/TWEAK ratio of children with obesity compared to controls. A possible explanation for this might be that this cytokine is involved in processes such as cell proliferation and differentiation (36), present in childhood growth. The protective role of TWEAK in obesity is not demonstrated in prepubertal children, although some studies observed an anti-inflammatory role of this cytokine in obese adults (25, 37).

Regarding CD163, this cytokine is higher in adults with obesity, and known as a biomarker of insulin resistance (23). Carolan et al. observed elevated CD163 in children with obesity, suggesting that it could be a biomarker to prioritize lifestyle intervention in childhood (38). In our study, CD163 serum levels were also higher in children with obesity compared to lean.

The present study is the first to observe a favorable effect of a 2-year lifestyle intervention program in prepubertal children who achieved a significant weight loss compared with those who did not. We observed expected higher levels of TWEAK, lower CD163/TWEAK ratio, and a more pronounced decrease of CD163 in the group of patients who achieved weight loss.

This study is the first one to observe the evolution of these parameters in prepubertal children, supporting the hypotheses that these cytokines may play a role in childhood obesity. One study found similar results in adults with severe obesity after weight loss achieved by bariatric surgery (25). Kazankov et al. observed a decrease in CD163 after 10 weeks of a lifestyle intervention in children with a mean age of 12 years (39).

This research has some limitations. It has a weak external validity because all subjects were only from one center. However, inclusion criteria for subjects are enough to guarantee a strong internal validity in our results. Unfortunately we don’t have estradiol, testosterone levels or TWEAK evolution of control group after two years of follow-up.

Our findings show that TWEAK and CD163 may be involved in the pathogenesis of obesity in prepubertal children. Childhood is a necessary stage for the prevention of consequences derived from obesity. This study opens an interesting line of research aimed at this objective.

## Data Availability Statement

The original contributions presented in the study are included in the article/supplementary material. Further inquiries can be directed to the corresponding author.

## Ethics Statement

The studies involving human participants were reviewed and approved by CEIC from FUNDACIÓ INSTITUT D’INVESTIGACIÓ I INNOVACIÓ PARC TAULÍ (I3PT). Written informed consent to participate in this study was provided by the participants’ legal guardian/next of kin.

## Author Contributions

AC, RC, and JG-C conceived the study. RC obtained informed consent and clinical variables. JV analyzed samples. MF and RE performed literature search. All authors were involved in writing the paper and had final approval of the submitted and published versions.

## Funding

This project was supported by a grant from Fundació Parc Taulí 2004104.

## Conflict of Interest

The authors declare that the research was conducted in the absence of any commercial or financial relationships that could be construed as a potential conflict of interest.

## Publisher’s Note

All claims expressed in this article are solely those of the authors and do not necessarily represent those of their affiliated organizations, or those of the publisher, the editors and the reviewers. Any product that may be evaluated in this article, or claim that may be made by its manufacturer, is not guaranteed or endorsed by the publisher.
